# COPA syndrome in an Icelandic family caused by a recurrent missense mutation in *COPA*

**DOI:** 10.1186/s12881-017-0490-8

**Published:** 2017-11-14

**Authors:** Brynjar O. Jensson, Sif Hansdottir, Gudny A. Arnadottir, Gerald Sulem, Ragnar P. Kristjansson, Asmundur Oddsson, Stefania Benonisdottir, Hakon Jonsson, Agnar Helgason, Jona Saemundsdottir, Olafur T. Magnusson, Gisli Masson, Gudmundur A. Thorisson, Adalbjorg Jonasdottir, Aslaug Jonasdottir, Asgeir Sigurdsson, Ingileif Jonsdottir, Vigdis Petursdottir, Jon R. Kristinsson, Daniel F. Gudbjartsson, Unnur Thorsteinsdottir, Reynir Arngrimsson, Patrick Sulem, Gunnar Gudmundsson, Kari Stefansson

**Affiliations:** 1 deCODE Genetics/Amgen, Inc, Sturlugata 8, 101, Reykjavik, Iceland; 2 Department of Respiratory Medicine and Sleep, Landspitali University Hospital, Reykjavik, Iceland; 3 Department of Anthropology, University of Iceland, Reykjavik, Iceland; 4 Department of Pathology, Landspitali University Hospital, Reykjavik, Iceland; 5 Department of Pediatrics, Landspitali University Hospital, Reykjavik, Iceland; 6 School of Engineering and Natural Sciences, University of Iceland, Reykjavik, Iceland; 7 Faculty of Medicine, University of Iceland, Reykjavik, Iceland; 8 Department of Genetics and Molecular Medicine, Landspitali University Hospital, Reykjavik, Iceland; 9 Department of Biochemistry and Molecular Biology, University of Iceland, Reykjavik, Iceland

**Keywords:** COPA syndrome, Lung disease, Arthritis, Immune dysregulation, Case report

## Abstract

**Background:**

Rare missense mutations in the gene encoding coatomer subunit alpha (*COPA*) have recently been shown to cause autoimmune interstitial lung, joint and kidney disease, also known as COPA syndrome, under a dominant mode of inheritance.

**Case presentation:**

Here we describe an Icelandic family with three affected individuals over two generations with a rare clinical presentation of lung and joint disease and a histological diagnosis of follicular bronchiolitis. We performed whole-genome sequencing (WGS) of the three affected as well as three unaffected members of the family, and searched for rare genotypes associated with disease using 30,067 sequenced Icelanders as a reference population. We assessed all coding and splicing variants, prioritizing variants in genes known to cause interstitial lung disease. We detected a heterozygous missense mutation, p.Glu241Lys, in the *COPA* gene, private to the affected family members. The mutation occurred de novo in the paternal germline of the index case and was absent from 30,067 Icelandic genomes and 141,353 individuals from the genome Aggregation Database (gnomAD). The mutation occurs within the conserved and functionally important WD40 domain of the COPA protein.

**Conclusions:**

This is the second report of the p.Glu241Lys mutation in *COPA*, indicating the recurrent nature of the mutation. The mutation was reported to co-segregate with COPA syndrome in a large family from the USA with five affected members, and classified as pathogenic. The two separate occurrences of the p.Glu241Lys mutation in cases and its absence from a large number of sequenced genomes confirms its role in the pathogenesis of the COPA syndrome.

**Electronic supplementary material:**

The online version of this article (10.1186/s12881-017-0490-8) contains supplementary material, which is available to authorized users.

## Background

Autoimmune interstitial lung, joint, and kidney disease (AILJK) is an immunodeficiency disorder identified in 2015 that impacts pulmonary, renal, and connective tissue function [1]. It is also termed COPA syndrome since it is caused by heterozygous missense mutations in the *COPA* gene (specifically in the WD40 domain of the coatomer subunit alpha). COPA syndrome is inherited under autosomal dominant mode of inheritance with variable expressivity. Most COPA syndrome patients present early in life, with lung biopsies showing follicular bronchiolitis. Additionally, pulmonary hemorrhage is often a presenting feature of the disease [1]. The *COPA* gene encodes the alpha subunit of the coatomer protein complex (COPI), a carrier complex required for retrograde protein trafficking from the Golgi to the endoplasmic reticulum (ER) [2]. The pathological mechanism of COPA syndrome is thought to consist of impaired return of proteins from the Golgi to the ER. This deficit of proteins is compensated for by increasing protein translation which in turn increases ER stress and results in abnormal cellular autophagy [2].

**Fig. 1:**
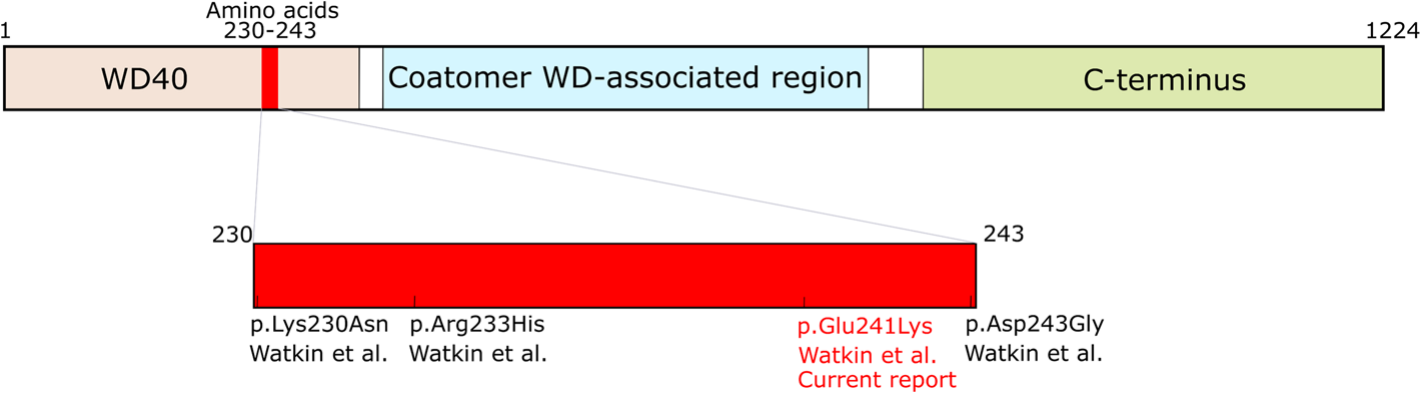
Illustration of the COPA protein. Previously reported mutations in *COPA* (Watkin et al., 2015) all cluster within a 14 amino acid stretch in the COPA protein. The mutation detected in the Icelandic pedigree is listed in red. The current report marks the second observation of the p.Glu241Lys mutation

To date, four missense mutations in *COPA* have been reported in a single publication in individuals within five families with COPA syndrome [2]. All four mutations cluster within a 14 amino acid stretch (residues 230-243) in the functionally important WD40 domain of the COPA protein (Fig. 1). WD-repeat proteins are made up of highly conserved repeating units and regulate important cellular functions, such as cell division, cell-fate determination, and vesicular trafficking and fusion [3–5]. The importance of these proteins is further highlighted by the fact that disruption of the gene that encodes the alpha-subunit of the COPI in yeast (RET1P), a protein containing four WD-40 repeated motifs, is lethal [6].

The development of follicular bronchiolitis is a common feature of COPA syndrome [1]. Follicular bronchiolitis is characterized by the presence of hyperplastic lymphoid follicles with germinal centers in the walls of small airways [7]. This histopathology can be seen in several clinical settings, such as immunodeficiency, connective tissue diseases, autoimmune diseases, and interstitial lung diseases in both children and adults.

**Fig. 2:**
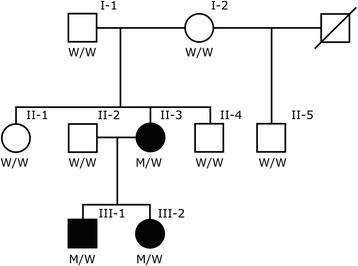
The three-generation family. Shown are genotypes of the *COPA* mutation (W: wild-type allele, M: mutated allele). Blackened symbols denote affected individuals, unblackened symbols denote unaffected individuals, and the slashed line denotes deceased. The genotypes of the siblings of the index case (II-3) were obtained with Sanger sequencing and the other six family members were whole-genome sequenced

In the current study, we analyzed an Icelandic family, with three members carrying the diagnosis of follicular bronchiolitis (Fig. 2). Six family members (including the three affected) were whole-genome sequenced with the aim of searching for rare sequence variants causing the condition.

## Case presentation

The index case of the study (II-3, b. 1978) first sought medical attention at the age of 32 because of exercise intolerance. She had previously been diagnosed with hay fever and juvenile arthritis and had no history of smoking. Lung biopsy taken at the age of 33 showed chronic bronchiolitis, multiple foci of lymphoid hyperplasia in the pulmonary interstitium and mild interstitial lymphoid infiltrate. Additionally, distal acinar emphysema and multifocal interstitial neuroendocrine cell hyperplasia was noted. These results lead to the histological diagnosis of follicular bronchiolitis (Additional file 1: Figure S1). The index case (II-3) had progressive dyspnea and respiratory tract infections over the next several years and required supplemental oxygen. Pulmonary function testing performed at age 43 showed severe restrictive lung disease with a very low diffusing capacity for carbon monoxide (DLCO) (Additional file 2: Table S1). She underwent a bilateral lung transplant at the age of 44. II-3 has two affected offspring, a son born in 1988 (III-1) and a daughter born in 1995 (III-2). III-1 was initially diagnosed with asthma at 11 years of age. Additionally, he had diffuse intra-alveolar hemorrhage and presented with arthritis of wrists, fingers, ankles and knees. He had an open lung biopsy at 13 years of age, which showed chronic bronchiolitis, multiple lymphoid follicles adjacent to terminal bronchioles and in the pulmonary interstitium and small peripheral cyst formations. This lead to the histological diagnosis of follicular bronchiolitis (Additional file 3: Figure S2).

Similar to his mother he experienced increasing exercise intolerance and recurrent respiratory infections over the next several years and required supplementary oxygen. At the age of 28, his pulmonary function had worsened considerably and showed severe obstructive lung disease, with hyperinflation and air-trapping along with very low DLCO (Additional file 2: Table S1). He received a bilateral lung transplant at 28 years of age, after we performed the whole-genome sequencing.

III-2 was diagnosed with juvenile rheumatoid arthritis at 18 months of age. From the age of 10, she had exercise intolerance, recurrent respiratory infections and a recurring skin condition. She had a lung biopsy at 13 years of age that showed chronic bronchiolitis as well as multiple lymphoid follicles adjacent to bronchioles and in the interstitium, which lead to her being histologically diagnosed with follicular bronchiolitis (Additional file 4: Figure S3). Additional autoimmune and rheumatological features for all three patients can be found in Additional file 5: Table S2 as well as a summary of the treatment they received. The CARE guidelines were followed in this case description.

In our analysis, we assessed all coding and splice variants prioritizing variants in genes known to cause interstitial lung disease (detailed description in Additional file 6: Supplementary Information). Before the analysis, the most compatible inheritance model was found to be autosomal dominant, since three individuals of both genders in subsequent generations were affected with the same disease. The parents of the index case are both Icelandic and have currently reached 75 and 72 years of age, respectively, and do not have a diagnosis of lung disease.

When searching for the causative genotype under an autosomal dominant mode of inheritance in the affected family members we filtered out mutations with MAF over 0.1% in Iceland (see Additional file 6: Supplementary Information and Additional file 7: Figure S4). For each of the remaining variants we checked if they were in genes known to cause human disease [8]. Whole-genome sequencing revealed a missense mutation (NM_001098398.1: c.721G > A / NP_004362.2: p.Glu241Lys) in the *COPA* gene, private to the three affected family members (Additional file 8: Table S3). The presence of the mutation in cases and its absence from other family members (including the two parents of the index case) was confirmed with Sanger sequencing, indicating a de novo mutation in the index case (Additional file 9: Table S4). Additionally, the mutation was absent from the genomes of 30,067 Icelanders who have been whole-genome sequenced to an average depth of at least 30× at deCODE genetics, and from 141,353 genomes/exomes publically available on the genome Aggregation Database (gnomAD) [9]. No other candidates, likely to explain the disease in the affected family members, were detected in our analysis.

The p.Glu241Lys mutation was the only coding or splice de novo mutation in the index case. In order to confirm that the mutation occurred de novo, we checked and confirmed the relationship between the index case and her two unaffected parents by haplotype sharing between them, as well as the transmission of rare variants. Additionally, by using haplotype sharing between the family members we found out that the de novo mutation was present on the paternal haplotype background of the index case. Whole-genome and Sanger sequencing of a blood sample from the father (I-1) both show no traces of the mutation, consistent with the mutation occurring de novo in the paternal germline of the index case (Additional file 9: Table S4).

The p.Glu241Lys mutation is a known pathogenic mutation, reported in five affected individuals with hereditary autoimmune-mediated lung disease and arthritis [2] from a family of Irish/Native American descent (Anthony Shum, personal communication). In vitro functional expression assays performed by Watkin et al. [2] showed that the mutation caused impaired binding to a dilysine reporter, suggesting that it causes a defect in retrograde transport from the Golgi to the endoplasmic reticulum. Table 1 lists the phenotypic overlap between these individuals and the ones described in the present study, which includes lung and joint disorders. No signs of kidney disease were observed in the two families.

**Table 1:** The reported phenotypes for two affected family members of Family C in Watkin et al. compared to the phenotypes seen in the affected Icelanders

	Index case (II-3)	Son (III-1)	Daughter (III-2)	Family C from Watkin et al. [2]
*COPA* mutation	p.Glu241Lys	p.Glu241Lys	p.Glu241Lys	p.Glu241Lys (2/2)
Sex	Female	Male	Female	Female (*N* = 2)
Age of onset	Child	11 years old	18 months	2 and 4 years old
Respiratory/ Lung	Lung restriction	Lung obstruction	–	Lung restriction / obstruction (2/2)
	Follicular bronchiolitis	Follicular bronchiolitis	Follicular bronchiolitis	Interstitial lung disease (2/2)
	Diffusion capacity defect	Diffusion capacity defect	Diffusion capacity defect	Diffusion capacity defect (2/2)
	Respiratory infections	Respiratory infections	Respiratory infections	
	Exercise intolerance	Exercise intolerance	Nail Clubbing	
		Pulmonary hypertension		
		Intra-alveolar hemorrhage		Pulmonary hemorrhage (1/2)
Kidney	–	–	–	–
Joints	Arthritis	Arthritis, joint pain	Arthritis	Arthritis, joint pain (2/2)
Serologies	↑ ANA (mild)	↑ ANA	↑ ANA	↑ ANA (1/2)
	↑ RF (mild)	↑ RF (very mild)	↑ RF	ANCA neg. (2/2)
	anti-CCP neg.	↑ anti-CCP (very mild)	↑ anti-CCP	
		ANCA neg.	ANCA neg.	
Immunoglobulins	↑ IgG (mild)	↑ IgG (mild), ↑ IgA (mild)	↓ IgG (total, IgG1 and IgG4), ↓ IgA	↑ IgG (1/2), ↑ IgA (1/2)
Therapy	Lung transplantation	Lung transplantation	Steroids	Steroids (2/2)
	Oxygen therapy	Oxygen therapy	Immunosuppressants	Immunosuppressants (2/2)
	Steroids	Steroids	Bronchodilators	
	Immunosuppressants	Immunosuppressants	Immunoglobulins	

## Discussion and conclusions

This report marks the second occurrence of the p.Glu241Lys mutation in cases with COPA syndrome. The mutation occurred de novo in the paternal germline of the index case and was inherited by her two affected offspring with lung and joint disease. The recurrence of the mutation in Iceland raises the question whether the genomic position (chr1:160,314,111, hg38) is highly mutable. In addition, as initially reported, all pathogenic *COPA* mutations cluster in a 14 amino acid interval of the WD40 domain. It remains to be understood if this area has a higher mutation rate than other regions of the gene or alternatively is the only region with a pathogenic potential. Recurrent de novo mutations in *ATP1A3* and *FGFR3* were previously reported to cause the majority of cases in alternating hemiplegia of childhood [10] and achondroplasia [11], respectively, which similarly lead to the speculation of a hypermutable sequence in those genes.

The p.Glu241Lys mutation was first reported in five affected individuals with COPA syndrome from a large family in the US (Family C) [2]. The US family included 55 individuals over six generations, where whole-exome sequencing was performed of five affected individuals and four unaffected members of the pedigree. All five affected individuals carried the p.Glu241Lys mutation in addition to two unaffected family members, indicating incomplete penetrance, although it is possible that clinical information on the two unaffected carriers was missing. In the current Icelandic pedigree, the mutation is fully penetrant and private to the three affected family members. Interestingly, both families carrying the p.Glu241Lys mutation have normal kidney function in contrast to the other four families (families A, B, D, and E) [2]. This suggests that the mutation does not have deleterious effects on renal function.

*COPA* is a highly constrained gene, with a large deficit in both missense (273 vs. 429, z-score: 3.69) and loss of function (0 vs. 54, pLI-score: 1.00) mutations [12, 13]. Furthermore, the mutation position (chr1:160,314,111) is highly conserved as indicated by its GERP [14] score of 5.62 (Additional file 8: Table S3). This means that predicted loss-of-function mutations (frameshift, stop, and essential splice) in *COPA* would have the same deleteriousness as missense mutations in the WD40 domain. This is consistent with functional results performed by Watkin et al. [2], showing that *COPA* missense mutations result in a defective COPA protein, although it remains uncertain whether the mutations produce disease through a dominant-negative or haploinsufficient effect [1].

To our knowledge, this is the second report of a pathogenic mutation in *COPA*. The p.Glu241Lys mutation detected in the current Icelandic pedigree is a de novo mutation. The fact that the mutation is absent from 176,040 unrelated individuals but present in two families with affected individuals confirms its role in the pathogenesis of COPA syndrome. To understand whether there is a mutational hotspot in the *COPA* gene, more cases of the newly defined COPA syndrome will have to emerge.

## Additional files

Additional file 1: Figure S1. Lung biopsy section from the index case (II-3) stained with hematoxylin and eosin. Mild interstitial lymphoid infiltrate and multiple foci of lymphoid hyperplasia. (DOCX 579 kb)

Additional file 2: Table S1. Pulmonary function test results from the three affected Icelanders with several years passing between the two tests. (DOCX 13 kb)

Additional file 3: Figure S2. Lung biopsy section from the son of the index case (III-1) stained with hematoxylin and eosin. Multiple lymphoid follicles in the pulmonary interstitium and adjacent to a terminal bronchiole (arrow). (DOCX 906 kb)

Additional file 4: Figure S3. Lung biopsy section from the daughter of the index case (III-2) stained with hematoxylin and eosin. Multiple lymphoid follicles in the interstitium and adjacent to airways (arrow). (DOCX 856 kb)

Additional file 5: Table S2. Additional clinical features. Autoimmune and rheumatological features as well as antibody titers for the three patients. The medications and general measures taken to treat the patients are listed. (DOCX 13 kb)

Additional file 6: Supplementary Information. Detailed description of methods (sample preparation, whole-genome sequencing, alignment, variant calling and annotation) and the genetic analysis performed in this study (Additional file 10: Table S5). (DOCX 23 kb)

Additional file 7: Figure S4. Variant filtering flowchart. We detected an average of 4,942,809 coding and non-coding variants per affected member of the family. Sixteen variants with MAF <0.1% in the Icelandic population (MAF derived from 30,067 Icelandic genomes) were shared by the three affected. Out of the 16 variants, one was private to the affected family members. (DOCX 41 kb)

Additional file 8: Table S3. *COPA* mutation. Summary of the causative mutation causing the lung disease in the Icelandic family. (DOCX 19 kb)

Additional file 9: Table S4. Sequencing data metrics for the *COPA* mutation. The call ratios of the *COPA* mutation for the index case, affected son and daughter, and unaffected husband, mother, and father from the WGS data. Additionally, the Sanger sequencing genotypes for the three siblings of the index case are listed. (DOCX 13 kb)

Additional file 10: Table S5. Coding variants with MAF < 0.1% in Iceland shared between all three affected family members. (DOCX 14 kb)

## Abbreviations

AILJK: Autoimmune interstitial lung, joint and kidney disease
COPA: Coatomer subunit alpha
COPI: Coatomer protein complex
DLCO: Diffusing capacity for carbon monoxide
ER: Endoplasmic reticulum
GERP: Genomic evolutionary rate profiling
gnomAD: Genome aggregation database
MAF: Minor allelic frequency
pLi: Probability of being loss-of-function intolerant
WGS: Whole-genome sequencing

## References

[1] Vece TJ, Watkin LB, Nicholas SK, Canter D, Braun MC, Guillerman RP (2016). Copa syndrome: a novel Autosomal dominant immune Dysregulatory disease. J Clin Immunol.

[2] Watkin LB, Jessen B, Wiszniewski W, Vece TJ, Jan M, Sha Y (2015). COPA mutations impair ER-Golgi transport and cause hereditary autoimmune-mediated lung disease and arthritis. Nat Genet.

[3] Eugster A, Frigerio G, Dale M, Duden R (2000). COP I domains required for coatomer integrity, and novel interactions with ARF and ARF-GAP. EMBO J.

[4] Neer EJ, Schmidt CJ, Nambudripad R, Smith TF (1994). The ancient regulatory-protein family of WD-repeat proteins. Nature.

[5] Li D, Roberts R (2001). WD-repeat proteins: structure characteristics, biological function, and their involvement in human diseases. Cell Mol Life Sci.

[6] Gerich B, Orci L, Tschochner H, Lottspeich F, Ravazzola M, Amherdt M (1995). Non-clathrin-coat protein alpha is a conserved subunit of coatomer and in Saccharomyces Cerevisiae is essential for growth. Proc Natl Acad Sci U S A.

[7] Tashtoush B, Okafor NC, Ramirez JF, Smolley L (2015). Follicular Bronchiolitis: a literature review. J Clin Diagn Res.

[8] McKusick VA (2007). Mendelian inheritance in man and its online version, OMIM. Am J Hum Genet.

[9] Lek M, Karczewski KJ, Minikel EV, Samocha KE, Banks E, Fennell T (2016). Analysis of protein-coding genetic variation in 60,706 humans. Nature.

[10] Heinzen EL, Swoboda KJ, Hitomi Y, Gurrieri F, Nicole S, de Vries B (2012). De novo mutations in ATP1A3 cause alternating hemiplegia of childhood. Nat Genet.

[11] Bellus GA, Hefferon TW, Ortiz de Luna RI, Hecht JT, Horton WA, Machado M (1995). Achondroplasia is defined by recurrent G380R mutations of FGFR3. Am J Hum Genet.

[12] ExAC. Exome Aggregation Consortium (ExAC) browser. 2015. http://exac.broadinstitute.org. Accessed 6 Jan 2016.

[13] Samocha KE, Robinson EB, Sanders SJ, Stevens C, Sabo A, McGrath LM (2014). A framework for the interpretation of de novo mutation in human disease. Nat Genet.

[14] Cooper GM, Stone EA, Asimenos G, NISC Comparative Sequencing Program, Green ED, Batzoglou S, et al. Distribution and intensity of constraint in mammalian genomic sequence. Genome Res. 2005;15:901–13. doi:10.1101/gr.3577405. 10.1101/gr.3577405 PMC1172034 15965027

