# Supplementary material for: COPA syndrome in an Icelandic family caused by a recurrent missense mutation in COPA
Source: BMC Med Genet. 2017 Nov 14;18:129. doi: 10.1186/s12881-017-0490-8 (PMC5686906; doi:10.1186/s12881-017-0490-8)
Supplement: Supplementary file 1 — Lung biopsy section from the index case (II-3) stained with hematoxylin and eosin. Mild interstitial lymphoid infiltrate and multiple foci of lymphoid hyperplasia. (DOCX 579 kb) [file 12881_2017_490_MOESM1_ESM.docx]

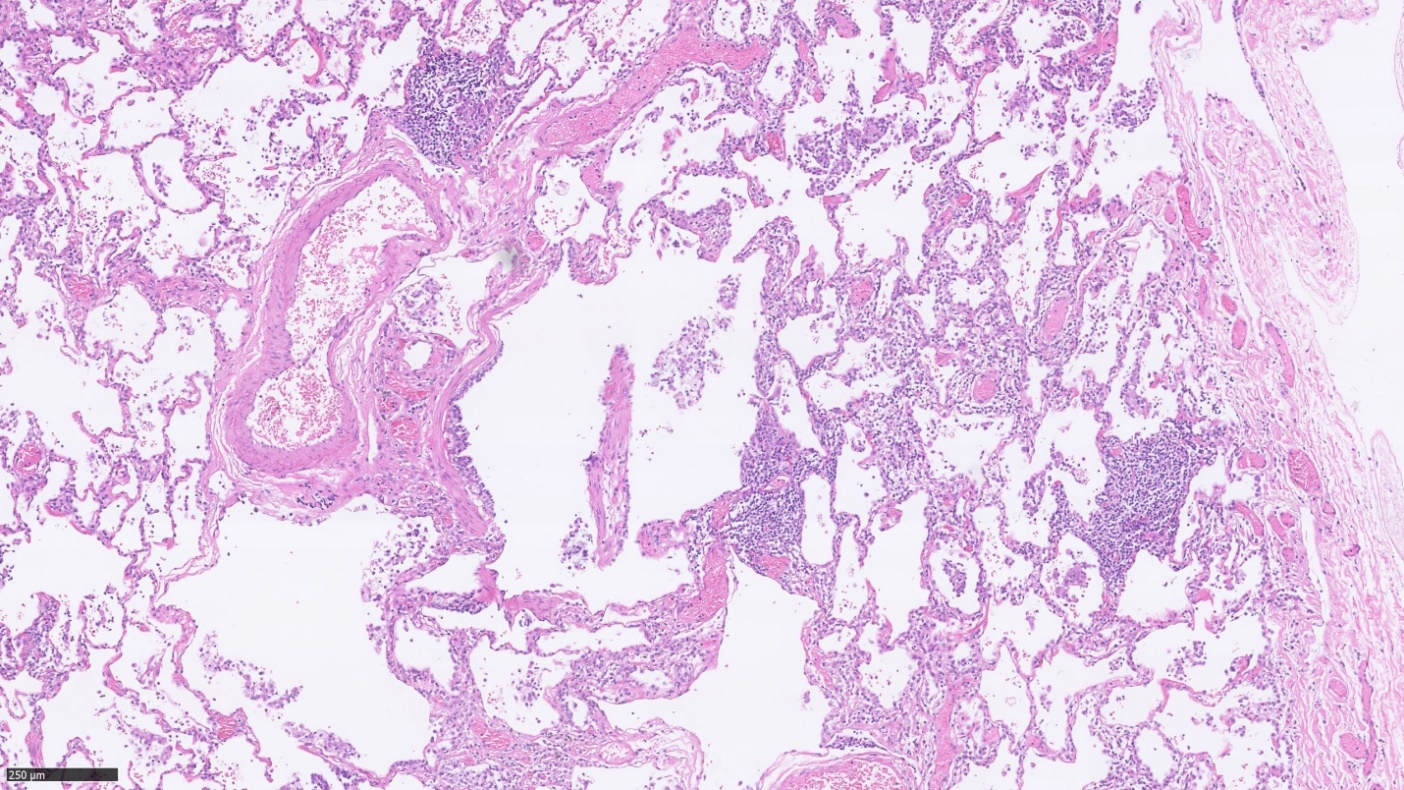


**Figure S1:** Lung biopsy section from the index case (II-3) stained with hematoxylin and eosin. Mild interstitial lymphoid infiltrate and multiple foci of lymphoid hyperplasia.
