# Supplementary material for: COPA syndrome in an Icelandic family caused by a recurrent missense mutation in COPA
Source: BMC Med Genet. 2017 Nov 14;18:129. doi: 10.1186/s12881-017-0490-8 (PMC5686906; doi:10.1186/s12881-017-0490-8)
Supplement: Supplementary file 2 — Pulmonary function test results from the three affected Icelanders with several years passing between the two tests. (DOCX 13 kb) [file 12881_2017_490_MOESM2_ESM.docx]

| **Table S1**: Pulmonary function test results from the three affected Icelanders with several years passing between the two tests. | | | | | | |
| --- | --- | --- | --- | --- | --- | --- |
|  | **Index case (II-3)** | | **Affected son (III-1)** | | **Affected daughter (III-2)** | |
| Age when tested | 35 | 43 | 16 | 28 | 9 | 21 |
| **Spirometry** |  |  |  |  |  |  |
| FVC | 2.64 (69%) | 1.52 (37%) | 4.13 (93%) | 3.51 (65%) | 1.98 (59%) | 2.17 (56%) |
| FEV1 | 2.11 (64%) | 1.32 (40%) | 3.27 (89%) | 2.02 (46%) | 1.77 (61%) | 1.92 (56%) |
| Ratio | 0.8 | 0.87 | 0.79 | 0.58 | 0.90 | 0.88 |
|  |  |  |  |  |  |  |
| **Lung diffusion** |  |  |  |  |  |  |
| DLCO | N/A (41%) | 1.6 (17%) | 6.7 (58%) | 2.54 (22%) | 7.9 (48%) | 3.17 (33%) |
|  |  |  |  |  |  |  |
| **Lung volume** |  |  |  |  |  |  |
| TLC | 3.85 (69%) | 2.38 (43%) | 5.67 (101%) | 6.99 (100%) | 3.12 (72%) | N/A |
| RV | 1.19 (71%) | 0.86 (48%) | 1.47 (123%) | 3.27 (196%) | 1.14 (105%) | N/A |
