# Supplementary material for: COPA syndrome in an Icelandic family caused by a recurrent missense mutation in COPA
Source: BMC Med Genet. 2017 Nov 14;18:129. doi: 10.1186/s12881-017-0490-8 (PMC5686906; doi:10.1186/s12881-017-0490-8)
Supplement: Supplementary file 4 — Lung biopsy section from the daughter of the index case (III-2) stained with hematoxylin and eosin. Multiple lymphoid follicles in the interstitium and adjacent to airways (arrow). (DOCX 856 kb) [file 12881_2017_490_MOESM4_ESM.docx]

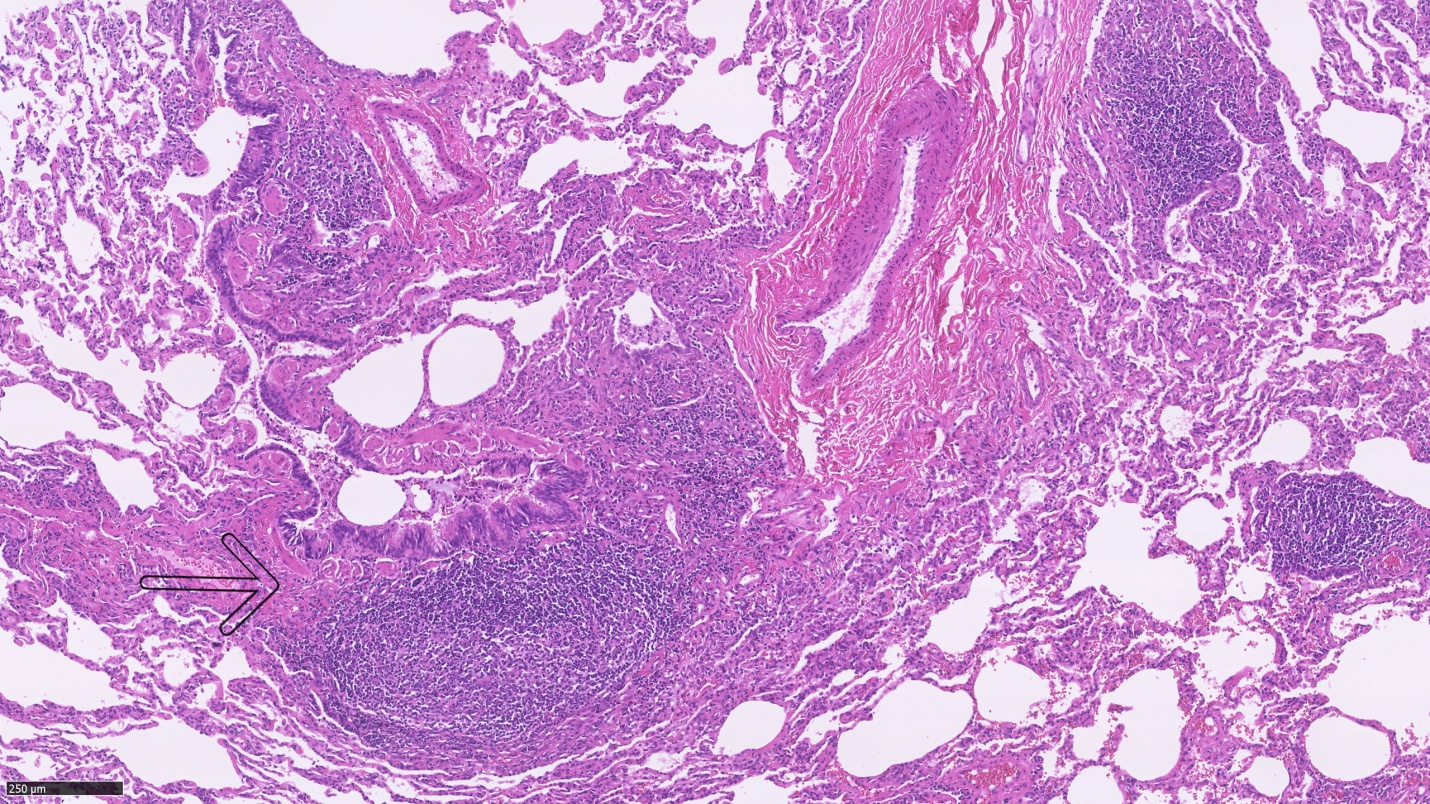


**Figure S3:** Lung biopsy section from the daughter of the index case (III-2) stained with hematoxylin and eosin. Multiple lymphoid follicles in the interstitium and adjacent to airways (arrow).
