# Supplementary material for: COPA syndrome in an Icelandic family caused by a recurrent missense mutation in COPA
Source: BMC Med Genet. 2017 Nov 14;18:129. doi: 10.1186/s12881-017-0490-8 (PMC5686906; doi:10.1186/s12881-017-0490-8)
Supplement: Supplementary file 5 — Additional clinical features. Autoimmune and rheumatological features as well as antibody titers for the three patients. The medications and general measures taken to treat the patients are listed. (DOCX 13 kb) [file 12881_2017_490_MOESM5_ESM.docx]

| **Table S2**: Additional clinical features. Autoimmune and rheumatological features as well as antibody titers for the three patients. The medications and general measures taken to treat the patients are listed. | | | |
| --- | --- | --- | --- |
|  | **Index case (II-3)** | **Affected son (III-1)** | **Affected daughter (III-2)** |
| **Autoimmune and rheumatological features**  **Antibody titers** | Arthritis (no erosions)  RF rheumaton (+)  ANA (+) | Arthritis (wrists, fingers, ankles, knees – no erosions).  RF rheumaton (-)  RF ELISA 4.2 (<4)  Anti-CCP 28 U/ml (<25)  ANA 1:300 | Juvenile rheumatoid arthritis  Skin rash, suspicion of erythema multiforme  RF rheumaton (+)  RF RAPA 1:160  RF ELISA 112 (<4)  - IgM > 383 (<25)  - IgG 75  Anti-CCP 1338 (>25)  ANA 1:300 |
| **Treatment** |  |  |  |
| Immunosuppression and inflammation | Steroids (i.v., p.o., inhaled)  Anti-inflammatory (NSAIDs)  Azathioprine  Mycophenolate mofetil | Steroids (p.o., inhaled)  Anti-inflammatory (NSAIDs)  Methotrexate  Mycophenolate mofetil  Azithromycin (immune modulation) | Steroids  Anti-inflammatory (NSAIDs)  Methotrexate  Salazopyrin  TNF inhibitors  Immunoglobulin  Mycophenolate mofetil |
| General | Bronchodilators  Repeated courses of antibiotics  Anti-acid medications  Lung transplantation | Bronchodilators  Repeated courses of antibiotics  Anti-acid medications  Acetylcystein  Oxygen therapy  Sildenafil  Lung transplantation | Bronchodilators  Repeated courses of antibiotics  Immunoglobulins  Anti-acid medications |
