# Supplementary material for: COPA syndrome in an Icelandic family caused by a recurrent missense mutation in COPA
Source: BMC Med Genet. 2017 Nov 14;18:129. doi: 10.1186/s12881-017-0490-8 (PMC5686906; doi:10.1186/s12881-017-0490-8)
Supplement: Supplementary file 6 — Detailed description of methods (sample preparation, whole-genome sequencing, alignment, variant calling and annotation) and the genetic analysis performed in this study (Additional file 10: Table S5). (DOCX 23 kb) [file 12881_2017_490_MOESM6_ESM.docx]

**Supplementary Information**

**Methods**

**Sample requisition and delivery**: Genomic DNA (gDNA) samples are requested for whole-genome sequencing (WGS) through an in-house laboratory information management system (LIMS). gDNA is registered (PN) and isolated by an in-house core facility (Biological Materials facility). Samples are delivered to the Genome Sequencing Laboratory in a barcoded 96 well tray format and stored at 4°C until use.

**Sample preparation using the TruSeq PCR-free method**: Samples are prepared for sequencing using the TruSeq PCR-free library preparation kits from Illumina. In short, 1 μg of gDNA, isolated from either frozen blood samples or buccal swabs, is fragmented to a mean target size of 300-400 bp using a Covaris E220 instrument. End repair, generating blunt ended fragments is done followed by size selection using different ratios of AMPure XP magnetic purification beads. 3’-Adenylation and ligation of indexed (96 dual indices) sequencing adaptors containing a T nucleotide overhang is performed, followed by AMPure purification. The quality and concentration of all sequencing libraries is assessed using the LabChip GX (96-samples) instrument from Perkin Elmer. Sequencing libraries are diluted to 3nM concentration and stored at −20 °C. Further quality control of sequencing libraries is done by multiplexing and pooling 96 samples (one 96-well tray) and sequencing each pool on an Illumina MiSeq instrument to assess optimal cluster densities, library insert size, duplication rates and library diversities. All steps in the workflow are monitored using our in-house LIMS, with barcode tracking of all samples and reagents.

**DNA whole-genome sequencing on HiSeqX**:

Sequencing libraries are hybridized/clustered to the surface of paired-end (PE) HiSeq X version 2.5 flow cells, using the Illumina cBot™ instrument. Each library is hybridized to a single lane on a flow cell, yielding in general >30X sequence coverage. Paired-end sequencing-by-synthesis (SBS) is performed on Illumina HiSeq X instruments with a read length of 2x150 cycles of incorporation and imaging. Real-time analysis involves conversion of image data to base-calling in real-time. Base calling files (BCL) are transferred to an in-house data storage for further secondary pipeline analysis. Monitoring of flow cell/sample registration, run performance/completion and data yield is done via the in-house LIMS.

**Whole-genome alignment**: Sequence reads were aligned to NCBI’s Build 38 of the human reference sequence using the Burrows-Wheeler Aligner (BWA), version 0.7.10[1]. Alignments were merged into a single BAM file and marked for duplicates using Picard 1.55. Only non-duplicate reads were used for the downstream analysis.

**Whole-genome variant calling and annotation**: Variants were called using version 2014.4-2-g9ad6aa8 of the Genome Analysis Toolkit (GATK)[2], reads were called with GATK using a multi-sample configuration. The effects of sequence variants were annotated using release 80 of the Variant Effect Predictor[3].

**Genetic Analysis**

We performed whole-genome sequencing (at the average sequencing depth of 30×) on six family members (I-1, I-2, II-3, II-4, III-1 and III-2) to search for a causative mutation in the family. In our analysis, we focused on rare variants (SNPs, indels) at coding and splicing regions, as annotated by the Variant Effect Predictor[4]. deCODE genetics’ large-scale whole-genome sequencing of the Icelandic population has created a large normative set, currently consisting of 30,067 Icelanders, which constitutes 10% of the population. We use this normative set to derive the minor allelic frequency (MAF) of sequence variants in Iceland. Additionally, we use the minor allelic frequency of sequence variants from over 4,000 individuals of other ethnicities sequenced at deCODE as well as publically available databases such as the genome Aggregation Database (gnomAD)[5], which is derived from 141,353 genomes and/or exomes.

In this study, we assessed all coding and splice variants prioritizing variants in genes known to cause interstitial lung disease. Before the analysis, the most compatible inheritance model was found to be autosomal dominant, since three individuals of both genders in subsequent generations were affected with the same disease. In addition, all other inheritance models were assessed (autosomal recessive, X-linked recessive and mitochondrial). We have no knowledge of consanguinity in the family.

When searching for the causative genotype under an autosomal dominant mode of inheritance in the affected family members we prioritized rare variants (MAF <0.1% in Iceland). For each of these rare variants we checked if they were in genes known to cause human disease[6]. Sixteen rare variants were shared by the index case (II-3) and her two affected offspring (III-1 and III-2, Additional file 10: Table S5). Out of the sixteen variants, two were private to the pedigree (with no other Icelandic carriers). One of the variants (NP_001274365.1: p.Glu494_Gln499dup) was in *COBL*, a gene with no known link to human disease. This variant was also seen in the unaffected mother of the index case (I-2). The other was a missense mutation (NM_001098398.1: c.721G>A / NP_004362.2: p.Glu241Lys) in the *COPA* gene, and private to the three affected family members (Additional file 8: Table S3).

Besides the p.Glu241Lys mutation, no other candidates, following an autosomal dominant mode of inheritance, were detected in our analysis. In addition, no rare or low frequency variants following autosomal recessive (homozygous or compound heterozygous) or X-linked modes of inheritance were detected in the genomes of the affected individuals that could explain their phenotypes. Furthermore, no evidence was found of any mitochondrial mutations of significance.

**References**

1. Li H, Durbin R. Fast and accurate short read alignment with Burrows-Wheeler transform. Bioinformatics. 2009;25:1754–60. doi:10.1093/bioinformatics/btp324.

2. McKenna A, Hanna M, Banks E, Sivachenko A, Cibulskis K, Kernytsky A, et al. The Genome Analysis Toolkit: a MapReduce framework for analyzing next-generation DNA sequencing data. Genome Res. 2010;20:1297–303. doi:10.1101/gr.107524.110.

3. McLaren W, Pritchard B, Rios D, Chen Y, Flicek P, Cunningham F. Deriving the consequences of genomic variants with the Ensembl API and SNP Effect Predictor. Bioinformatics. 2010;26:2069–70. doi:10.1093/bioinformatics/btq330.

4. McLaren W, Gil L, Hunt SE, Riat HS, Ritchie GRS, Thormann A, et al. The Ensembl Variant Effect Predictor. Genome Biol. 2016;17:122. doi:10.1186/s13059-016-0974-4.

5. Lek M, Karczewski KJ, Minikel E V, Samocha KE, Banks E, Fennell T, et al. Analysis of protein-coding genetic variation in 60,706 humans. Nature. 2016;536:285–91. doi:10.1038/nature19057.

6. McKusick VA. Mendelian Inheritance in Man and Its Online Version, OMIM. Am J Hum Genet. 2007;80:588–604. doi:10.1086/514346.
