# Supplementary material for: COPA syndrome in an Icelandic family caused by a recurrent missense mutation in COPA
Source: BMC Med Genet. 2017 Nov 14;18:129. doi: 10.1186/s12881-017-0490-8 (PMC5686906; doi:10.1186/s12881-017-0490-8)
Supplement: Supplementary file 7 — Variant filtering flowchart. We detected an average of 4,942,809 coding and non-coding variants per affected member of the family. Sixteen variants with MAF <0.1% in the Icelandic population (MAF derived from 30,067 Icelandic genomes) were shared by the three affected. Out of the 16 variants, one was private to the affected family members. (DOCX 41 kb) [file 12881_2017_490_MOESM7_ESM.docx]

**
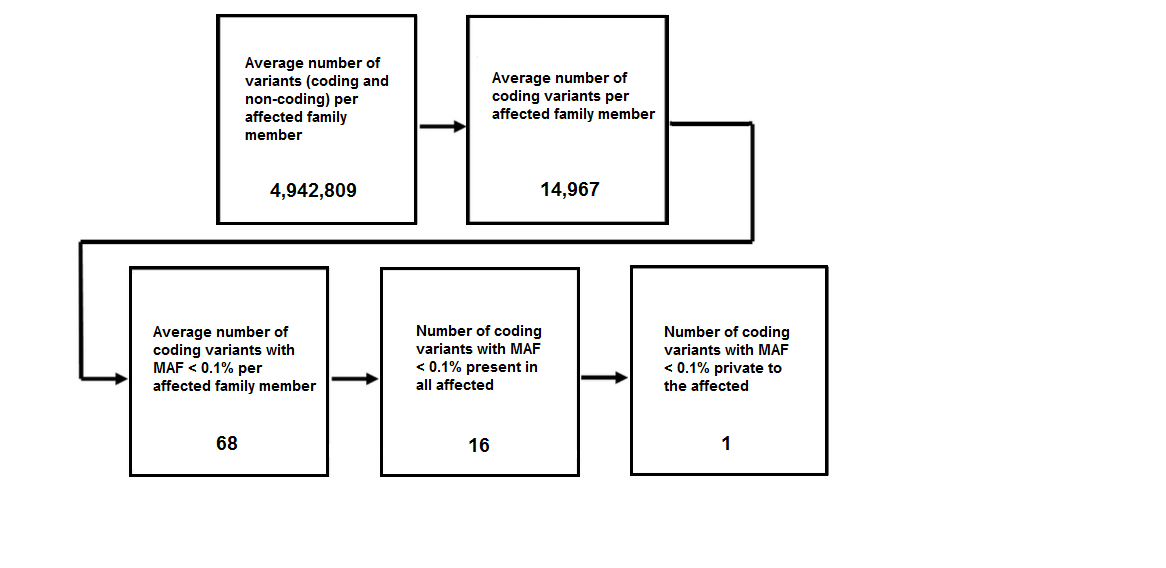
Figure S4**: Variant filtering flowchart. We detected an average of 4,942,809 coding and non-coding variants per affected member of the family. Sixteen variants with MAF <0.1% in the Icelandic population (MAF derived from 30,067 Icelandic genomes) were shared by the three affected. Out of the sixteen variants, one was private to the affected family members.
