# Supplementary material for: COPA syndrome in an Icelandic family caused by a recurrent missense mutation in COPA
Source: BMC Med Genet. 2017 Nov 14;18:129. doi: 10.1186/s12881-017-0490-8 (PMC5686906; doi:10.1186/s12881-017-0490-8)
Supplement: Supplementary file 8 — COPA mutation. Summary of the causative mutation causing the lung disease in the Icelandic family. (DOCX 19 kb) [file 12881_2017_490_MOESM8_ESM.docx]

| **Table S3**: Summary of the causative mutation causing the lung disease in the Icelandic family. | |
| --- | --- |
| ***Gene:*** | ***COPA*** |
| **Chromosomal position (hg38/build 38)** | chr1:160314111 |
| **Variant type** | missense |
| **Variant genotype** | heterozygous in 3/3 affected de novo in index case |
| **HGVSc. (RefSeq transcript)** | c.721G>A (NM_004371.3) |
| **HGVSp. (RefSeq transcript)** | p.Glu241Lys (NP_004362.2) |
| **Exon number / Total exon number** | 9/33 |
| **Gene size** | 1224 amino acids |
| **Allelic frequency** | Absent from 30,067 Icelanders (WGS) Absent from 141,353 individuals from gnomAD (WGS/WES) |
| **ExAC constraint score*** | (3.69) constrained gene for missense |
| **GERP**** | (5.62) highly conserved position |
| **SIFT** | deleterious (0) |
| **PolyPhen-2** | possibly damaging (0.868) |
| **Disease in literature** | Autoimmune interstitial lung, joint and kidney disease  (OMIM: #616414, ClinVar: RCV000180778.3) |
| **Variant classification** | Pathogenic  Reported in Watkin et al.[1] and OMIM (*601924.0003) |

**COPA* is a highly constrained gene, with a large deficit in observed vs. expected number of missense (273 vs. 429, z-score: 3.69 [95^th^ percentile]) and loss of function (0 vs. 54, pLI-score: 1.00 [99^th^ percentile]) mutations from 60,706 exomes in ExAC[2, 3].

**The high level of conservation of the mutation position is indicated by its GERP score of 5.62 (maximum genome-wide score is 6.17)[4].

**References**

1. Watkin LB, Jessen B, Wiszniewski W, Vece TJ, Jan M, Sha Y, et al. COPA mutations impair ER-Golgi transport and cause hereditary autoimmune-mediated lung disease and arthritis. Nat Genet. 2015;47:654–60. doi:10.1038/ng.3279.

2. ExAC. Exome Aggregation Consortium (ExAC) browser. 2015. http://exac.broadinstitute.org. Accessed 6 Jan 2016.

3. Samocha KE, Robinson EB, Sanders SJ, Stevens C, Sabo A, McGrath LM, et al. A framework for the interpretation of de novo mutation in human disease. Nat Genet. 2014;46:944–50. doi:10.1038/ng.3050.

4. Cooper GM, Stone EA, Asimenos G, Comparative N, Program S, Green ED, et al. Distribution and intensity of constraint in mammalian genomic sequence. 2005;:901–13.
