# Supplementary material for: COPA syndrome in an Icelandic family caused by a recurrent missense mutation in COPA
Source: BMC Med Genet. 2017 Nov 14;18:129. doi: 10.1186/s12881-017-0490-8 (PMC5686906; doi:10.1186/s12881-017-0490-8)
Supplement: Supplementary file 9 — Sequencing data metrics for the COPA mutation. The call ratios of the COPA mutation for the index case, affected son and daughter, and unaffected husband, mother, and father from the WGS data. Additionally, the Sanger sequencing genotypes for the three siblings of the index case are listed. (DOCX 13 kb) [file 12881_2017_490_MOESM9_ESM.docx]

| **Table S4**: Sequencing data metrics for the *COPA* mutation. The call ratios of the *COPA* mutation for the index case, affected son and daughter, and unaffected husband, mother, and father from the WGS data. Additionally, the Sanger sequencing genotypes for the three siblings of the index case are listed | | | | | | |
| --- | --- | --- | --- | --- | --- | --- |
| **Family member** | **Identity** | **Affected** | **Call Ratio** | **Depth (x)** | Ref. (C) / Alt. (T) | **Genotype** |
| **Index case** | II-3 | Yes | 0.49 | 41 | 21 / 20 | CT |
| **Husband** | II-2 | No | 0.0 | 38 | 38 / 0 | CC |
| **Affected son** | III-1 | Yes | 0.52 | 60 | 29 / 31 | CT |
| **Affected daughter** | III-2 | Yes | 0.46 | 41 | 22 / 19 | CT |
| **Mother** | I-2 | No | 0.0 | 31 | 31 / 0 | CC |
| **Father** | I-1 | No | 0.0 | 38 | 38 / 0 | CC |
| **Brother*** | II-4 | No | - | - | - | CC |
| **Sister*** | II-1 | No | - | - | - | CC |
| **Half-brother* (maternal)** | II-5 | No | - | - | - | CC |

*Genotype acquired with Sanger sequencing

To assess the quality of the COPA mutation, we checked the sequencing depth for the chromosomal position as well as the quality of the reads (Supplementary Table 3). Sanger sequencing confirmed the presence of the mutation in all three affected (indicated by the CT genotype) and its absence from other family members (indicated by the CC genotype) and we can therefore conclude that the mutation occurred *de novo* in the index case.
