# Supplementary material for: COPA syndrome in an Icelandic family caused by a recurrent missense mutation in COPA
Source: BMC Med Genet. 2017 Nov 14;18:129. doi: 10.1186/s12881-017-0490-8 (PMC5686906; doi:10.1186/s12881-017-0490-8)
Supplement: Supplementary file 10 — Coding variants with MAF < 0.1% in Iceland shared between all three affected family members. (DOCX 14 kb) [file 12881_2017_490_MOESM10_ESM.docx]

| **Table S5**: Coding variants with MAF < 0.1% in Iceland shared between all three affected family members. | | | | | |
| --- | --- | --- | --- | --- | --- |
| **Chr. pos. (hg38)** | **Ref. allele** | **Alt. allele** | **Impact** | **Gene** | **HGVSc. / HGVSp.** |
| chr1:160314111 | C | T | MODERATE | *COPA* | NM_004371.3:c.721G>A NP_004362.2:p.Glu241Lys |
| chr7:51043460 | G | GTCCTGGTCACTGCAGTCT | MODERATE | *COBL* | NM_015198.3:c.1311_1328dup  NP_056013.2:p.Glu437_Gln442dup |
| chr21:26480299 | G | A | MODERATE | *CYYR1* | NM_052954.2:c.307C>T NP_443186.1:p.His103Tyr |
| chr8:133113469 | C | G | HIGH | *TG* | NM_003235.4:c.7620C>G  NP_003226.4:p.Tyr2540Ter |
| chr11:95185291 | T | C | MODERATE | *SESN3* | NM_144665.3:c.727A>G  NP_653266.2:p.Ile243Val |
| chr3:48580643 | C | T | MODERATE | *COL7A1* | NM_000094.3:c.4990G>A  NP_000085.1:p.Gly1664Arg |
| chr7:140567204 | C | T | MODERATE | *DENND2A* | NM_015689.3:c.1661G>A  NP_056504.3:p.Arg554Gln |
| chr20:56517108 | G | C | HIGH | *RTFDC1* | NM_001283036.1:c.647-1G>C |
| chr17:12994838 | G | A | MODERATE | *ELAC2* | NM_001165962.1:c.1835C>T NP_001159434.1:p.Ala612Val |
| chr2:44326839 | TTAA | T | MODERATE | *PREPL* | NM_001042385.2:c.1430_1432delTTA  NP_001035844.1:p.Ile477del |
| chr18:33943120 | G | C | MODERATE | *NOL4* | NM_001198546.1:c.1295C>G  NP_001185475.1:p.Ala432Gly |
| chr19:55518327 | T | TCC | HIGH | *SSC5D* | NM_001144950.1:c.4051_4052insCC  NP_001138422.1:p.Pro1352LeufsTer4 |
| chr14:60153157 | C | T | MODERATE | *DHRS7* | NM_016029.2:c.415G>A NP_057113.1:p.Gly139Ser |
| chr1:150961006 | G | A | MODERATE | *SETDB1* | NM_001145415.1:c.2944G>A  NP_001138887.1:p.Ala982Thr |
| chr1:205058645 | C | G | MODERATE | *CNTN2* | NM_005076.3:c.469C>G  NP_005067.1:p.Pro157Ala |
| chr1:152218646 | C | T | MODERATE | *HRNR* | NM_001009931.2:c.2983G>A  NP_001009931.1:p.Gly995Ser |
